# Identifying context factors explaining physician's low performance in communication assessment: an explorative study in general practice

**DOI:** 10.1186/1471-2296-12-138

**Published:** 2011-12-13

**Authors:** Geurt Essers, Sandra van Dulmen, Chris van Weel, Cees van der Vleuten, Anneke Kramer

**Affiliations:** 1 Department of Primary & Community Care, Radboud University Nijmegen Medical Centre, (Geert Groteplein 21), Nijmegen, (6525 EP), The Netherlands; 2 Netherlands Institute for Health Services Research NIVEL, (Otterstraat 118-124), Utrecht, (3513 CR), The Netherlands; 3 Department of Educational Development and Research, Maastricht University, (Universiteitssingel 60), Maastricht, (6229 ER), The Netherlands

## Abstract

**Background:**

Communication is a key competence for health care professionals. Analysis of registrar and GP communication performance in daily practice, however, suggests a suboptimal application of communication skills. The influence of context factors could reveal why communication performance levels, on average, do not appear adequate. The context of daily practice may require different skills or specific ways of handling these skills, whereas communication skills are mostly treated as generic. So far no empirical analysis of the context has been made. Our aim was to identify context factors that could be related to GP communication.

**Methods:**

A purposive sample of real-life videotaped GP consultations was analyzed (N = 17). As a frame of reference we chose the MAAS-Global, a widely used assessment instrument for medical communication. By inductive reasoning, we analyzed the GP behaviour in the consultation leading to poor item scores on the MAAS-Global. In these cases we looked for the presence of an intervening context factor, and how this might explain the actual GP communication behaviour.

**Results:**

We reached saturation after having viewed 17 consultations. We identified 19 context factors that could potentially explain the deviation from generic recommendations on communication skills. These context factors can be categorized into doctor-related, patient-related, and consultation-related factors.

**Conclusions:**

Several context factors seem to influence doctor-patient communication, requiring the GP to apply communication skills differently from recommendations on communication. From this study we conclude that there is a need to explicitly account for context factors in the assessment of GP (and GP registrar) communication performance. The next step is to validate our findings.

## Background

Communication is a key competence for health care professionals. Good communication determines the quality of care [1-4] and is highly valued by patients [5]. In the GP Specialty Training, the training of communication skills is an essential part [6]. There are indications, however, that the effects of such communication skills training for GP registrars are limited [7-10], although a recent study shows some improvement is possible [11]. Many registrars, however, find it difficult to apply acquired communication skills when working in daily practice [12]. Furthermore, the communication performance of experienced GPs, on average, does not appear to be adequate either [10,13,14].

Various explanations have been given for the low scores on communication skills. Firstly, it has been contended that the transfer is hampered by the separation of training and practice [15,16]. The setting of the training institute, using role play as the main teaching method, is too different from everyday clinical experiences in the setting of daily practice. There is evidence that communication training programmes, that are aligned to daily practice, have resulted in more and long term positive effects [17,18]. Secondly, a number of authors have pointed at the generic nature of recommendations on communication and instruments that are used to assess professionals' performance. The transfer of skills may be compromised even more due to the teaching of generic skills, while, in reality, GPs need to adjust their approach constantly to the specific context. Thirdly, the assumption that communication skills are generic and can be assessed as such may be unjustified [19-22]. As a consequence, however, all consultations are treated as if they were the same, whereas, in daily practice, GPs need to adjust their approach to the individual person presenting with a specific problem.

In the past few years, several researchers have pointed out that context factors on different levels influence communication in health care [23-26]. The influence of context factors could reveal why GP communication performance levels do not appear adequate, and moreover, could also provide an explanation for the limited effects of communication skills training for GP registrars, as they may play a vital role in allowing transfer to take place. Context factors range from a micro-level (patient and doctor characteristics) to meso- and macro-levels (organizational and societal features). According to Durning et al. [27] "context (1) comprises interacting factors that add to the meaning of something that exists or occurs in an environment, and (2) allows for change in that meaning as information is added over time." This definition points to the wide variability within consultations and the dynamic environment in which communication has to take place. In the assessment of communication skills, these factors have been mentioned as possibly interacting in the communication process, but so far no empirical analysis of how these factors are to be taken into account has been made [28].

If it is true that the context is a determining factor for the actual communication GPs display, this could lead to deviations from the recommendations on communication, as captured in assessment instruments [20]. Insight may be gained by observing communication of GPs in their natural work setting of daily practice [29]. There it can be examined if, how, and under which contextual conditions, the communication deviates from the recommendations. Our aim was to identify apparent context factors that could be related to GP communication. We were interested in GP communication behaviour that deviates from the generic criteria used in communication skills assessment [13]. And, if this occurs, whether it can be explained or justified by a particular context factor. As a first step towards accounting for context factors in communication assessment, we performed an explorative, qualitative study observing the communication in daily general practice consultations.

## Methods

Three researchers, each with different backgrounds (GP, communication researcher, and communication trainer), independently observed and analysed the same set of videotaped real-life GP consultations. For this, a purposive set of consultations (N = 17) was selected from a database of videotaped consultations of Dutch GPs, which were recorded as part of a video-observation study performed by NIVEL in 2007 - 2008 [30]. Selection criteria for the sample were: 1) a broad range of complaints or problems presented (different ICPC codes having a high prevalence in general practice) and 2) a variety of GPs, with an even distribution of male and female GPs. By including a broad range of health problems representative for general practice, we aimed to increase the chance to detect as many different context factors as possible, including the content of the problem.

### Observational framework

We used the MAAS-Global as the generic communication skills framework for our observations [28]. The MAAS-Global is a validated observation and assessment instrument, that serves as a guideline for patient-centred medical communication [31]. It is widely used in undergraduate medical and GP specialty training in the Netherlands [32]. The MAAS-Global consists of 13 generic communication items that can be rated from 0 ('absent') to 6 ('excellent'). Each item has three or four sub-items referring to criterion behaviour (see Additional file 1).

### Analysis

During data collection, we focused on the moments in the consultation where the GP's communication fell below the criteria for good communication as defined in the MAAS-Global (items scoring 'badly' or 'insufficiently'). After observing a consultation, each researcher reflected on the question whether the GP indeed performed poorly or whether the communication behaviour could be explained or justified, considering an observed context factor. These moments in the consultations were noted for further analysis. In a consensus meeting, we subsequently compared and analysed our notes and reflections, and discussed the possibly underlying context factors. A context factor was appointed by affirmative answers to the next questions: "Would the communicative behaviour of the GP have to be different if this context factor was not present?" and "Is the communicative behaviour of the GP (or the absence of it) adequate or logical in this context?". The alleged context factors were listed, aiming at completeness as well as consensus. This way, low scores on the MAAS-Global were related to 'adequate' or 'inadequate' professional performance.

We started analysing and discussing eight consultations in this manner, as this number is mentioned to control for case-specific aspects in communication assessment [13,31]. Subsequently, for practical reasons, we observed sets of three new consultations until no new context factors were identified. Saturation was reached after 17 consultations.

### Ethical regulations

The study was performed according to Dutch privacy legislation. The privacy regulation was approved by the Dutch Data Protection Authority. All participating GPs and patients signed an informed consent form before the recording of the consultation. According to Dutch legislation, approval by a medical ethics committee was not required for this observational study.

## Results

We found 19 context factors in GP consultations that could be related to low scores on the MAAS-Global. Table 1 lists the communication behaviour that was absent or deviating, indicated per MAAS-Global item, and the inferred context factors. The context factors could be categorized into doctor-related, patient-related, and consultation-related factors (Table 2). We will discuss our findings in more detail under these category headings.

**Table 1:** GP behaviour observed using MAAS-Global and context factors inferred by inductive reasoning^1^

MAAS-items	Observed GP communication behaviour	Inferred context factors
1. Opening	○ opening and establishing contact are very short ○ talks about patient's social and/or family circumstances ○ refers to prior contacts with patient or family ○ gives meaningful (non-)verbal signs of understanding ○ names patients communication pattern	- GP knows patient and his/her social context - GP knows patients' medical history - specific patient verbal behaviour *(e.g. patient is shy and tacit)* - GP knows patients' way of communicating - consultation in a series based on protocol (initiative by GP)

2. Follow-up consultation	○ does not name earlier complaints or management ○ does not ask about adherence ○ does not ask about course of complaint ○ goes to physical examination quickly (not asking explorative questions) ○ explicitly states that he follows the protocol	- GP knows patients' medical history - follow-up consultation in a series *(e.g. check up for blood pressure)* - consultation in a series based on protocol (initiative by GP) - consultation in preventive care (initiative by GP; there are no complaints) - specific patient verbal behaviour *(e.g. patient tells about adherence spontaneously)*

3. Request for help	○ does not name request for help ○ does not complete exploring request for help	- GP knows patients' way of communicating - consultation in preventive care (initiative by GP) - consultation based on protocol (initiative by GP) - specific patient verbal behaviour *(e.g. patient states wishes and expectations very clearly)*

4. Physical Examination (PE)	○ does not explain PE ○ does not give instructions (or only very brief) ○ does not explain or announce what is to be done (in simple PE)	- patient knows PE from prior consultations - patient has a disease (diagnosis) or (recurrent) problem known to both GP and patient - characteristics of physical examination

5. Diagnosis	○ does not do any diagnosing ○ does not name findings or diagnosis ○ names patient's health behaviour ○ refers to prior interview/diagnosis	- patient has a disease (diagnosis) or (recurrent) problem known to both GP and patient - patient is also treated by other provider - GP knows patient and his/her social context - GP knows patients' medical history - diagnosed problem is mainly psychosocial or psychiatric - specific patient non-verbal behaviour *(e.g. patient states diagnosis authoritatively, leaning forward)* - specific patient verbal behaviour *(e.g. patient tells extensively about medical history)*

6. Management	○ does not share decision on management ○ does not discuss alternatives ○ does not react to cues on psychosocial problems ○ does not discuss feasibility and adherence ○ does not discuss consulting with other provider ○ refers to management by co-provider ○ names patient's health behaviour ○ seems to anticipate on intermediate effects before next encounter	- patient is also treated by other provider - GP knows patients' medical history - specific patient non-verbal behaviour *(e.g. patient takes control)* - specific patient verbal behaviour *(e.g. patient tells about adherence)* - patient has a disease (diagnosis) or (recurrent) problem known to both GP and patient - the problem urgently needs medical care - first consultation in a series

7. Consultation closure	○ does not ask general evaluative question ○ does not check perspective for the time being ○ expresses hope that patient will benefit from consultation	- GP knows patient and his/her social context - specific patient non-verbal behaviour *(e.g. patient seems anxious)*

8. Exploration	○ does not react to cues on psychosocial problems ○ does not explore within patient's frame of reference ○ does not explore expectations or request for help	- GP knows patients' medical history - GP knows patients' way of communicating - GP knows patient and his/her social context - specific patient non-verbal behaviour *(e.g. patient seems impatient and puts pressure on GP)* - specific patient verbal behaviour *(e.g. patient presents physical complaints extensively and states wishes and expectations clearly)* - the problem urgently needs medical care - patient has a disease (diagnosis) or (recurrent) problem known to both GP and patient - consultation in preventive care (initiative by GP) - consultation based on protocol (initiative by GP)

9. Emotions	○ does not ask for emotions ○ does not reflect feelings	- GP knows patients' way of communicating - GP knows patient and his/her social context - specific patient verbal behaviour - diagnosed problem is easily solved

10. Providing Information	○ does not discuss consulting with other provider ○ does not announce or categorize information ○ refers to management by co-provider ○ uses authority and experience in providing information ○ invites patient to look into computer together	- GP knows patients' way of communicating - GP knows patient and his/her social context - patient is also treated by other provider - specific patient verbal behaviour *(e.g. patient anxiously asks many questions; patient uses medical jargon)* - specific patient non-verbal behaviour *(e.g. patient takes control)*

11. Summarizing	○ does not summarize	- specific patient verbal behaviour *(e.g. patient uses medical jargon)* - specific patient non-verbal behaviour *(e.g. patient takes control)* - diagnosed problem is easily solved

12. Structuring	○ sequence is not always logical ○ time spending is not balanced ○ does not/hardly announce phases ○ addresses more persons than patient alone, divides time adequately ○ assesses and structures involvement of other person(s)	- patient is familiar with (physical) examination (PE) - GP is very experienced - diagnosed problem is mainly psychosocial - there is more than one person (patient) present - specific patient verbal behaviour *(patient anxiously asks many questions)*

13. Empathy	○ does not express empathy in brief verbal responses	- GP knows patients' way of communicating - GP knows patient and his/her social context - specific patient verbal behaviour *(patient seems anxious)* - specific patient non-verbal behaviour *(e.g. patient takes control)* - diagnosed problem is easily solved

**^1 ^**communication behaviour and context factors are only listed; bullets and hyphens at the same height do not have a specific relationship.

**Table 2:** Context factors in GP consultations affecting communication process

doctor-related factors	
1.	doctor knows patient and his social context

2.	doctor knows patients' medical history

3.	doctor knows patients' way of communicating

4.	doctor is very experienced

**patient-related factors**	

5.	specific patient verbal behaviour

6.	specific patient non-verbal behaviour

7.	patient is also treated by other provider

8.	patient has a disease (diagnosis) or (recurrent) problem known to both doctor and patient

9.	patient is familiar with (physical) examination (PE)

**consultation-related factors**	

10.	single consultation

11.	first consultation in a series

12.	follow-up consultation in a series

13.	consultation in a series based on protocol (initiative by doctor)

14.	consultation in preventive care (initiative doctor)

15.	problem is mainly psychosocial

16.	diagnosed problem is easily solved

17.	problem urgently needs medical care

18.	more than one person (patient) present

19.	characteristics of physical examination

### Doctor-related factors

In 14 of the 17 consultations we observed the patient and GP discussing the patient's social and/or family circumstances (e.g. a patient who had recently had to move to a smaller house; a patient with a partner who has a serious health condition), or referring to prior contacts (e.g. in a consultation with a child that was taciturn and very difficult to engage). The communication in these consultations continued in a free and easy way, without much exploration of the patient's background. This social exchange usually took place at the start of the consultation (see Table 1). We considered the GP's knowledge of the patient and knowledge of the way the patient communicates to be influential context factors.

A related factor seemed to be the prior knowledge the GP had of the patient's medical history: generally the GP referred to a prior episode, or connected the current problems to the patient's medical history. This contrasted to the consultations where the GP seemed to have no prior knowledge of the current health problem the patient presented (e.g. reason for a referral to psychotherapy).

Moreover, more experienced GPs seemed to know what they were asking for, used fewer questions, applied the skill 'Structuring' more loosely, and without losing key information performed adequately on a medical level. Therefore we considered GP experience a relevant context factor as well.

### Patient-related factors

We observed patients who, at the beginning of the consultation, unsolicited, detailed and clearly, stated their health problem and related needs, preferences and expectations. The GP's response in these cases was restricted to a few additional clarifications or a very short history taking, prior to proceeding to the physical examination (PE). We also observed a patient who persevered in asking questions - out of anxiety or as a security check. This seemed to affect the GP's communication, leading to a focus on answering the questions and providing reassurance, but also to a decrease in expressed empathy. We combined these observations into one patient-related context factor: 'specific patient verbal behaviour'.

Another context factor was related to the patient's non-verbal presentation: incessant coughing, or severe paleness were informative symptoms that did not require more than perfunctory additional questions, before the GP decided on further diagnostic and therapeutic actions. Instead of summarizing, we observed the GP reacting directly to these presentations. In another consultation we saw the patient leaning forward and putting his arm on the GP's desk - seemingly emphasizing the importance of his verbal message. No menace was meant, but, in reaction, the GP did not further explore the patient's statements and proceeded to comply with his needs. In all cases, patient's behaviour seemed to influence GP's structuring behaviour, leading to an adaptation to the specific patient behaviour rather than sticking to the logical sequence of phases. Therefore, we considered 'specific non-verbal patient behaviour' to be a separate context factor.

In addition a context factor was inferred from cases where other professionals were involved in the treatment. We observed that these consultations focussed on questions on management that were important to the patient, while the diagnostic phase was partially or totally absent. For example, a female patient presented doubts on an upcoming operation, to which the GP responded by trying to reassure the patient and explain the goal and reasons for the operation.

Furthermore, we observed consultations in which the GP and the patient discussed the management of a health problem, but no history was taken. From this we concluded to be a context factor that the health problem was known to both of them. And from the observation of a patient who started to roll up his sleeve for his blood pressure check-up, without any prior instructions from the GP, we inferred that he must have been familiar with the procedure. Thus, we considered the patient's familiarity with the PE a context factor as well.

### Consultation-related factors

We observed a difference between follow-up and preventive consultations - initiated by the GP - on the one hand, and on the other hand consultations, in which the initiative to attend mainly lay with the patient. The former mostly were part of a chronic disease protocol (e.g. hypertension), to which the GP in one case explicitly referred. Here, the initiative came from the GP, whereupon the patient mostly agreed to attend, not necessarily having a problem. These consultations differed essentially from single consultations, first consultations in a series, and other follow-up consultations, in which the patient presented with a problem and the GP had to explore and find out what the patient required.

Also specific aspects of the presented problem were inferred as consultation-related factors. In dealing with complaints that were easily solved (e.g. removing cerumen or a suture), we saw the GP not going into emotions. We inferred that, as these complaints usually have little emotional impact, there is no need for the GP to discuss emotions. Problems needing urgent help were considered a context factor as well, as they tend to lead to direct action. In one consultation we observed a patient probably having suffered a TIA, for which the GP took action without exploring the patient's request for help. On the other hand, with a patient who presented problems in coping with her divorce, and problems with her son, we saw the GP expressing a lot of empathy and discussing the patient's feelings, but also losing the structure in the consultation. From this we considered psychosocial problems to be a context factor too.

The characteristics of the PE also seemed to make a difference. We observed that the PE could be simple or complex, invasive or superficial, leave room for social talk or require full attention of the GP. The absence of an explanation or only a very brief instruction in one case, in contrast to extensive instructions and explanations in another, led us to the inference that characteristics of the PE could be considered a context factor as well.

Finally, the number of persons present influenced the communication process. In these cases we saw the GP strive to divide their attention to those present and to involve everyone in the consultation process according to their role. This communication behaviour is not mentioned in the MAAS-Global.

### Context factors interacting in complex ways

Sometimes two or three context factors seemed to work synergistically. We saw an unexpected combination of doctor-related and patient-related factors, and characteristics of the PE, in a consultation with a female patient presenting for a routine check-up of her vaginal ring. The patient only briefly greeted the GP before proceeding to the examination room to undress. Before, during and after the examination patient and doctor chatted lightly, only once interrupted by a "You're OK" from the GP. Clearly, after many previous check-ups, the GP was able to perform the invasive, intimate examination without explaining, instructing, or even without announcing - without being disrespectful.

## Discussion

Our results show that in routine GP consultations, several context factors can be identified that - as a single factor or synergistically - clarify why GPs deviate from the recommended communication behaviour. In these consultations, in the judgement of experienced observers, a low score on communication skills items of the MAAS-Global is still accompanied by adequate professional performance. Several of the context factors we found point to the essence of general practice, such as continuity of care, a systems approach, prevention, treating (minor) ailments and problems with a psychosocial background [33-35].

We reported to have reached saturation after 17 consultations, because we did not find any new context factor in the last set of five consultations we observed, which is an acceptable criterion for this purpose [36]. Also in other explorative, qualitative research, the number of 17 consultations seems to fall within acceptable limits for saturation to be reached [37,38].

We found context factors that may explain GP's low scores on communication. These empirical results find theoretical resonance by looking at communication as goal-oriented behaviour. In the conceptual model by Feldman-Stewart [25], the communication process is directed by the goals each of the participants have - within the specific context that they are acting in. Other authors also pointed to the relevance of each of the participants goals for the communication process in the consultation [26,39,40]. If goals are modulated by the specific context and communication is goal-oriented, then context factors should explicitly play a role in the assessment of GP communication performance.

The relationship between the presence of a context factor and the communication behaviour of the GP, as we found it, is a logical one. If, for instance, the initiative for a consultation lies with the GP, it seems logical that there is no exploration of the patient's request for help. Obviously, if asked for the reason for the encounter, the patient would reply: "I'm here because you asked me to". However, the patient may still have questions concerning the goal of the consultation or the treatment he is receiving. Therefore, although the initiative for the consultation lies with the GP and there is no request for help from the patient, it does not discharge the GP from exploring questions that the patient may have. Similarly, in the case of an easily solved problem, like removing cerumen, it seems logical not to go into emotions. Nevertheless, the GP needs to stay attentive of emotions that may arise despite the simplicity of the complaint. Thus, context factors may explain why certain communication behaviour is absent, but they never justify its absence in all circumstances. This clearly reflects the dynamic way 'context' is to be understood [27].

In this study we restricted ourselves to identify context factors that are visible on a micro-level, but we did not look for context factors acting at meso- or macro-levels (organizational, demographical, political), that may also play a role [23]. For instance, the identification of preventive and follow-up consultations as a context factor may reflect the use of clinical practice guidelines that can be considered a context factor on a macro-level [41]. The fact that in the Dutch health care system the GP has a fixed patient list and acts as a gatekeeper for specialist care [42] is a societal context factor that may have contributed to the identification of doctor-related factors like 'doctor knows the patient and his social context', and the patient-related context factor 'patient is also treated by other provider'. In our view, these are important context factors on a micro-level, made possible by the position of the GP in the health care system. Thus, we do not claim to have found all context factors that are relevant to the communication in daily GP practice. Other research methods may shed light on the existence of contextual factors at other levels.

Not only did we observe communication skills being absent, but we also saw GPs exert communication behaviour not mentioned in the MAAS-Global (Table 1). Occasionally this was specifically related to a context factor: the fact that two or more persons were present elicited specific structuring behaviour, such as 'dividing time and attention adequately to all present', and 'involving those present adequately in their role'. In other consultations we observed the GP making use of their authority, or expressing hope that the consultation would benefit the patient, or naming patient's health behaviour, which directly seemed to affect the patient's understanding of their situation. We also saw GPs making use of their computer to inform patients on their health status. These are relevant communication skills that should be used to update the MAAS-Global.

Our findings may have implications for communication programmes in the GP specialty training. From what we found, it seems that the way generic communication assessment instruments are used does not suffice to justly assess communication performance in general practice. Moreover, training programmes should be organized around different types of consultations and should take into account that patients can be treated by other providers and know what is going to happen. The focus should be on the flexibility and creativity with which future GPs handle their communication skills. The application of communication skills in different contexts can be seen as working a mixing table: in a specific context, some channels are set to zero and others are maximized, all the time being ready to adapt to changes in the context. Future research could be directed at finding consensus on the ways communication patterns should adapt to context factors, and should focus on how to take the presence or absence of context factors into account in the assessment of GP communication behaviour.

### Strengths and limitations

By using real-life GP consultations, the ecological validity of our findings is strengthened. The different backgrounds and experience of the researchers add to this.

However, the method that we used can be considered a limitation of this study as it allowed us to find context factors at a micro-level, but not at other levels. We inferred context factors from low item scores on the MAAS-Global. Implicitly, this may suggest that a) only low scores are context-dependent, and b) high MAAS-Global scores represent a gold standard for communication. These implications are not intended. Firstly, high item scores may also lead to identification of context factors. However, in order to find explanations for GP communication performance that was less than expected, we logically focussed on low item scores. Secondly, other ways of analyzing communication behaviour can reveal very adequate communication patterns in experienced GPs that were not seen before [43].

As we did not select on age, gender or socio-economical class, the sample contained various patients with different ages and gender. However, a proportionate representation of patients from lower class or different ethnic origin was not seen. The behaviour stemming from different ethnic or cultural backgrounds can also be considered 'specific patient behaviour' to which the doctor needs to respond. Apart from this, the sample we saw seemed to be a fair representation of the consultations daily seen in GP practice [30].

## Conclusions

In this study, we found several context factors that may explain why the GP scored low on communication items of an assessment tool, yet displayed adequate professional performance. By identifying these context factors, we may have created a perspective to solve the limitations of generic communication assessment.

Explicitly including the identified context factors in communication training and assessment may be an elegant way to do justice to the complexity, diversity and specifics of daily general practice, and at the same time to not lose the importance of mastering separate communication skills.

## Competing interests

The study was funded by the SBOH Foundation, employer of Dutch General Practitioner Registrars and funder of the national specialty training for General Practitioners. There are no competing interests.

## Authors' contributions

All authors contributed to the concept and design of the study. GE, SvD and AK carried out the observations, and analyzed and interpreted the data. All authors commented on and approved the final manuscript.

## Pre-publication history

The pre-publication history for this paper can be accessed here:

http://www.biomedcentral.com/1471-2296/12/138/prepub

## Supplementary Material

Additional file 1 **MAAS-Global rating list for doctor-patient communication skills**. Overview of items and sub-items of the MAAS-Global. Click here for file
