# Supplementary material for: Identifying context factors explaining physician's low performance in communication assessment: an explorative study in general practice
Source: BMC Fam Pract. 2011 Dec 13;12:138. doi: 10.1186/1471-2296-12-138 (PMC3262758; doi:10.1186/1471-2296-12-138)
Supplement: Additional file 1 — MAAS-Global rating list for doctor-patient communication skills. Overview of items and sub-items of the MAAS-Global. [file 1471-2296-12-138-S1.DOC]

# Additional file 1

### **Title: MAAS-Global rating list for doctor-patient communication skills.**

Description: Overview of items and sub-items of the MAAS-Global. Items are scored on a scale from 0-6. Items 2 and 4 can be rated ‘not applicable’ additionally.

| **Communication skills for each separate phase**   1. **Introduction**   • giving the patient room to tell his story  • general orientation on the reason for visit  • asking about other reasons for visit   1. **Follow-up consultation**   • naming previous complaints, requests for help and management plan  • asking about adherence to management plan  • asking about the course of the complaint   1. **Request for help**   • naming requests for help, wishes or expectations  • naming reasons that prompted the patient to come now  • completing exploring request for help   1. **Physical examination**   • instructions to the patient  • explanation of what is being done  • treating the patient with care and respect   1. **Diagnosis**   • naming findings and diagnosis ⁄ hypothesis  • naming causes or the relation between findings and diagnosis  • naming prognosis or expected course  • asking for the patient’s response   1. **Management**   • shared decision making, discussing alternatives, risks and benefits  • discussing feasibility and adherence  • determining who will do what and when  • asking for patient’s response   1. **Evaluation of consultation**   • general question  • responding to requests for help  • perspective for the time being | **General communication skills**   1. **Exploration**   • exploring requests for help, wishes or expectations  • exploring patient’s response to information given within patient’s frame of reference  • responding to non-verbal behaviour and cues   1. **Emotions**   • asking about ⁄ exploring feelings  • reflecting feelings (including nature and intensity)  • sufficiently throughout the entire consultation   1. **Information giving**   • announcing, categorizing  • in small quantities, concrete explanations  • understandable language  • asking whether the patient understands   1. **Summarizations**   • content is correct, complete  • concise, rephrased  • checking  • sufficiently throughout the entire consultation   1. **Structuring**   • logical sequence of phases  • balanced division of time  • announcing (history taking, examination, other phases)   1. **Empathy**   • concerned, inviting and sincerely empathetic in intonation, gesture and eye contact  • expressing empathy in brief verbal responses |
| --- | --- |
